# Analyzing (3-Aminopropyl)triethoxysilane-Functionalized Porous Silica for Aqueous Uranium Removal: A Study on the Adsorption Behavior

**DOI:** 10.3390/molecules29040803

**Published:** 2024-02-09

**Authors:** Kegang Wei, Chin-Pao Huang

**Affiliations:** 1 Aquatic Chemistry Lab, Civil and Environmental Engineering Department, University of Delaware, Newark, DE 19711, USA; huang@udel.edu; 2 Jiangxi Copper Technology Institute Co., Ltd., Nanchang 330000, China

**Keywords:** uranium(VI), APTES-functionalized porous silica, surface complex formation, adsorption energy

## Abstract

This study synthesized (3-aminopropyl)triethoxysilane-functionalized porous silica (AP@MPS) to adsorb aqueous uranium (U(VI)). To comprehensively analyze the surface properties of the AP@MPS materials, a combination of SEM, BET, XPS, NMR, and zeta potential tests were conducted. The adsorption experiments for U(VI) revealed the rapid and efficient adsorption capacity of AP@MPS, with the solution condition of a constant solution pH = 6.5, an initial U(VI) concentration of 600 mg × L^−1^, a maximum U(VI) capacity of AP@MPS reaching 381.44 mg-U per gram of adsorbent, and a removal rate = 63.6%. Among the four types of AP@MPS with different average pore sizes tested, the one with an average pore size of 2.7 nm exhibited the highest U(VI) capacity, particularly at a pH of 6.5. The adsorption data exhibited a strong fit with the Langmuir model, and the calculated adsorption energy aligned closely with the findings from the Potential of Mean Force (PMF) analysis. The outcomes obtained using the Surface Complex Formation Model (SCFM) highlight the dominance of the coulombic force ΔG^0^_coul_ as the principal component of the adsorption energy (ΔG^0^_ads_). This work garnered insights into the adsorption mechanism by meticulously examining the ΔG^0^_ads_ across a pH ranging from 4 to 8. In essence, this study’s findings furnish crucial insights for the future design of analogous adsorbents, thereby advancing the realm of uranium(VI) removal methodologies.

## 1. Introduction

Nanostructured materials have emerged as advanced adsorbents for aqueous uranium recovery, gaining substantial attention post-2000s due to the improved methodologies involved. Their full development, especially for challenging contexts like nuclear power plant wastewater and seawater, still needs to be completed. Among these materials, mesoporous silica (MPS) is exceptional for uranium recovery adsorbents. MPS boasts surface silanol groups for easy modification with organic molecules like TEOS (tetraethyl orthosilicate) and TMOS (tetramethyl orthosilicate). It also offers adjustable pore sizes, high surface areas, and low environmental toxicity, making it ideal for advanced adsorbent design.

Noteworthy studies include Yang’s exploration of ion-imprinted mesoporous silica, showing notable uranium selectivity in acidic environments [1]. Khan et al. successfully immobilized U(VI) and Pb(II) with silica decorated with magnetite nanorods [2], while Gunathilake et al. examined amidoxime-modified mesoporous silica, showcasing excellent uranium recovery in seawater [3].

In the 2000s, renewed interest in nuclear energy post-2010 led the U.S. Department of Energy to focus on a new generation of uranium sorbents [4,5,6]. Amino-terminated adsorbents drew attention for their intriguing uranium adsorption effects [7], leading to growing interest in amino-functionalized porous silica materials [4,5].

Prior studies on these materials have mainly focused on aspects like their maximum capacity [8], selectivity [9], and removal efficiency [10,11]. Some have proposed interaction mechanisms between uranium and adsorbents theoretically. Examples include Vukovic et al. using density functional theory (DFT) at the B3LYP level to assess uranium–amidoxime bonding [12] and Li et al. introducing the Potential of Mean Force (PMF) model to simulate changes in adsorption energy at the adsorbent/solution interface [13].

However, certain studies have detected uranium on adsorbent surfaces without fully explaining the adsorption process. For instance, Abney et al. employed X-ray photoelectron spectroscopy to reveal uranium-adsorbent chelation motifs, conflicting with the related density functional theory work [14], and Comarmond et al. provided ATR FT-IR spectral data indicating the U(VI) adsorption onto TiO_2_ [15].

Many questions remain unanswered: How can positively charged U(VI) adsorb onto an electropositive surface? What components constitute adsorption energy? Addressing these gaps requires further exploration, including understanding an adsorbent’s electrical properties and uranium speciation. Determining the adsorption energy across various pH values allows for the assessment of energy [16,17], which can be further analyzed into components such as chemical energy (ΔG^0^_chem_), coulombic force (ΔG^0^_coul_), solvation energy (ΔG^0^solv), and lateral interaction energy (ΔG^0^_lat_). This study’s results may serve as a valuable reference for future investigations.

Table 1 details relevant acronyms and the substances they represent.

## 2. Results and Discussion

### 2.1. Surface Characteristics of Absorbents

#### 2.1.1. Physical Surface Characterization

**Figure 1:**
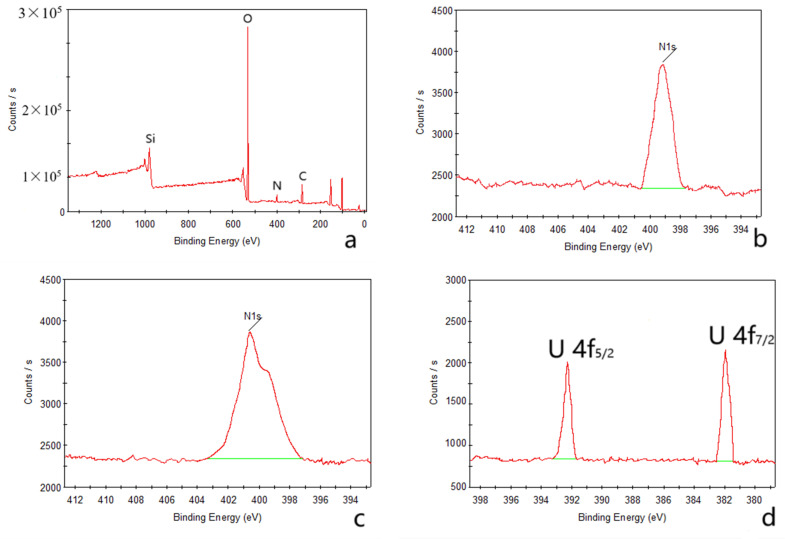
XPS test results for AP@MPS-9.2d; full survey spectra before adsorption (**a**), N1s spectra before (**b**) and after (**c**) adsorption, U 4f spectra (**d**).

**Figure 2:**
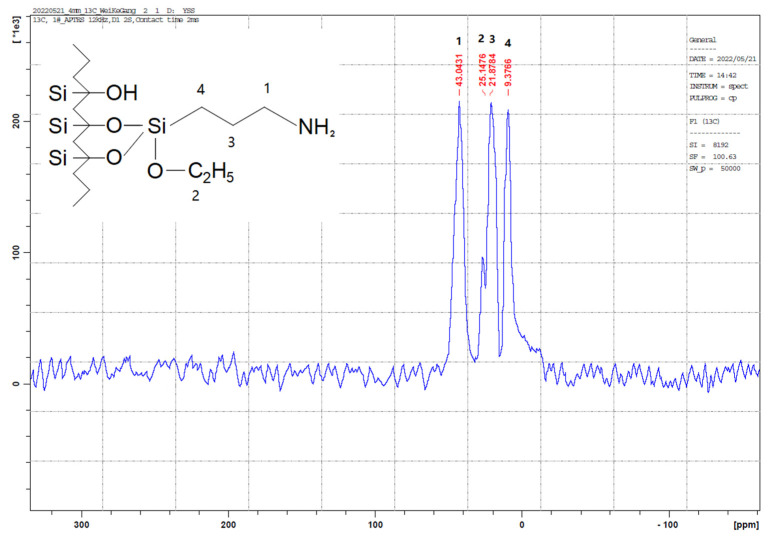
CP/MAS ^13^C nuclear magnetic resonance (NMR) spectra of AP@MPS, a total of four ^13^C signals detected.

**Figure 3:**
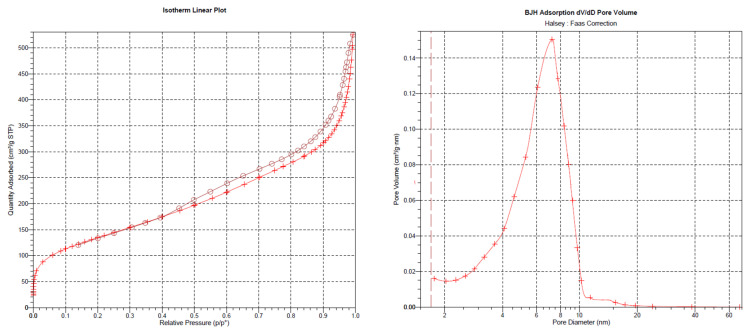
The BET test result of AP@MPS-6.3d; circle and + symbols represent desorb and adsorb process, respectively.

**Figure 4:**
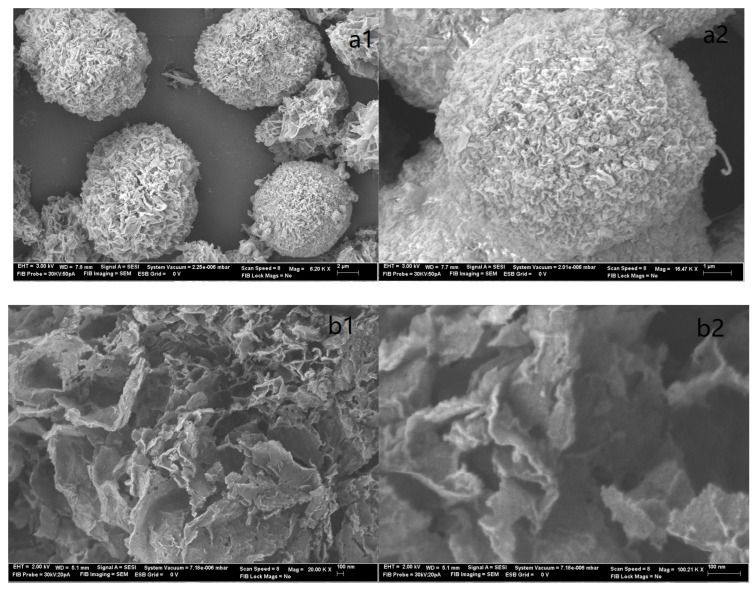
The SEM images of AP@MPS displaying (**a1**,**a2**) a low-magnification view (**on top**) and (**b1**,**b2**). a high-magnification view (**on bottom**).

The outcomes of the XPS analysis are presented in Figure 1. The XPS graph of the AP@MPS reveals an N1s peak at around 399.5 eV, confirming the successful grafting of APTES groups onto the MPS surface. After the uranium adsorption, AP@MPS shows two peaks at 381.9 eV and 392.2 eV in the XPS survey spectrum (Figure 1d). The two peaks are due to U 4f_5/2_ and U 4f_7/2_, respectively, and the spin-orbit split close to 10.3 eV (Figure 1d). After uranium adsorption, the N1s survey spectrum shifted by about 1 eV (Figure 1c) to the left, indicating the binding energy of the nitrogen in the -NH_2_ group increased by 1 eV, which means the electron density of the nitrogen atom decreased. The CP/MAS ^13^C NMR analysis can be found in Figure 2. Combining the XPS and NMR results substantiates the successful grafting of APTES onto the MPS surface. Table 2 provides the BET data for the samples, while Figure 3 illustrates the BET curve of AP@MPS-6.3d. Notably, other adsorbents with varying average pore sizes exhibit similar BET data. This implies that AP@MPS possesses a consistent and narrow pore distribution. Furthermore, the BET data highlight the adsorbents’ substantial surface area. Figure 4 displays SEM images of AP@MPS, revealing its circular-like appearance. The particle size of AP@MPS ranges from 1 to 10 µm. The images show that this kind of adsorbent’s surface is covered in creases and that the pores correspond to the creases between the aggregated silicon nanoparticles. However, these pores are not discernible in Transmission Electron Microscopy (TEM) images.

#### 2.1.2. Surface Electrical Properties

**Figure 5:**
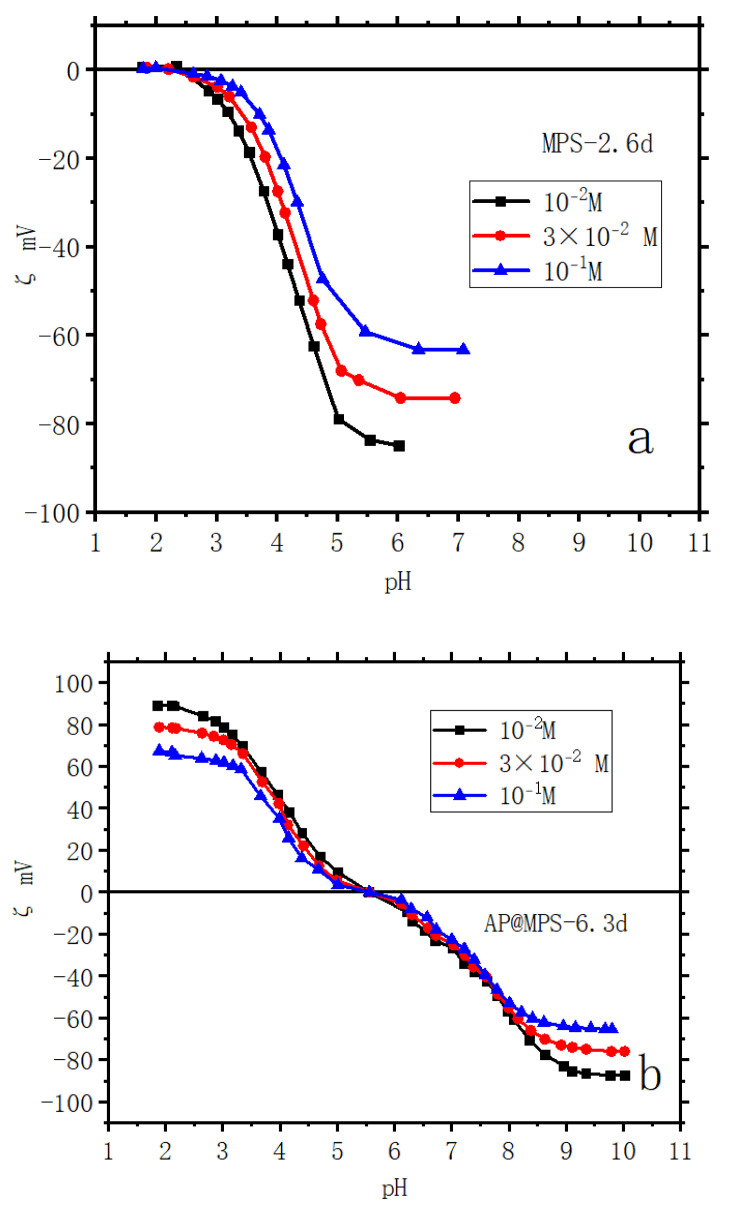
Zeta potential of samples: (**a**) MPS-2.6d; (**b**) AP@MPS-6.3d; the weight of adsorbent is 2 g∙L^−1^; ionic strengths are 10^−1^ M, 3 × 10^−2^ M, and 10^−2^ M with KClO_4_ as electrolyte; contact time is 24 h.

**Figure 6:**
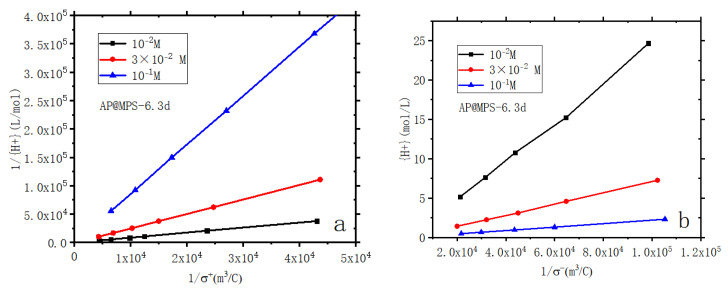
Linearized plots of surface acidity of AP@MPS-6.3d, (**a**) amino groups; (**b**) silica Groups. Experimental conditions: [W] = 2 g × L^−1^; ionic strengths = 10^−1^ M, 3 × 10^−2^ M, and 10^−2^ M in KClO_4_; contact time = 24 h.

**Figure 7:**
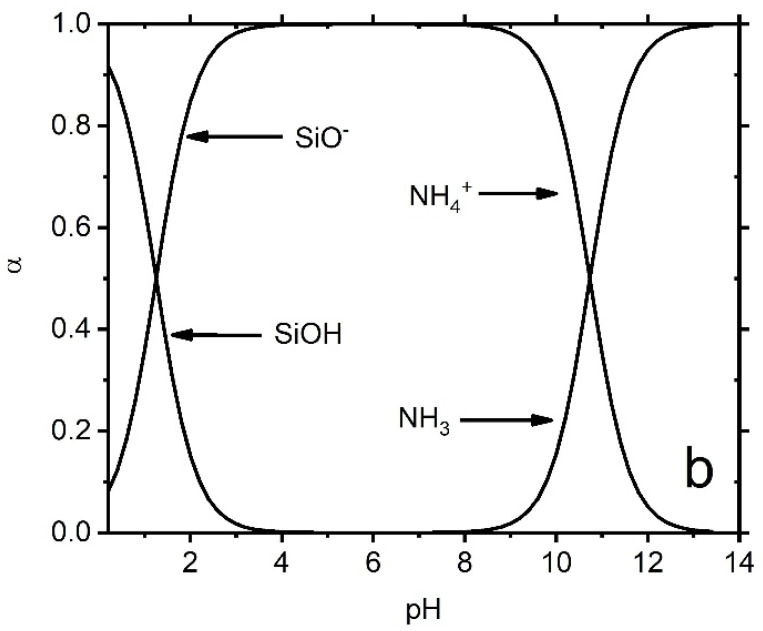
The specification of surface functional groups is on AP@MPS-9.2. The surface acidity of MPS is brought by two functional groups, namely SiOH/SiO^−^ and R-NH_n−1_/RNH_n_, which are Bronsted acids and bases.

The zeta potential curves presented in Figure 5 offer a comprehensive view of the surface electrical characteristics of the adsorbents across various ionic strengths. The determination of surface acidity is exhaustively explained in Ref. [18]. For surface acidity diagrams of AP@MPS-6.3d, refer to the illustrative depictions in Figure 6 and Figure 7. From the results of the BET analysis (Table 2) and from the zeta potential properties (Figure 5), we observe each couple of samples with similar pore sizes also have similar surface electrical properties.

### 2.2. Studied Adsorption Parameters: Equilibrium Time and Total Pore Volume

#### 2.2.1. Equilibrium Time

**Figure 8:**
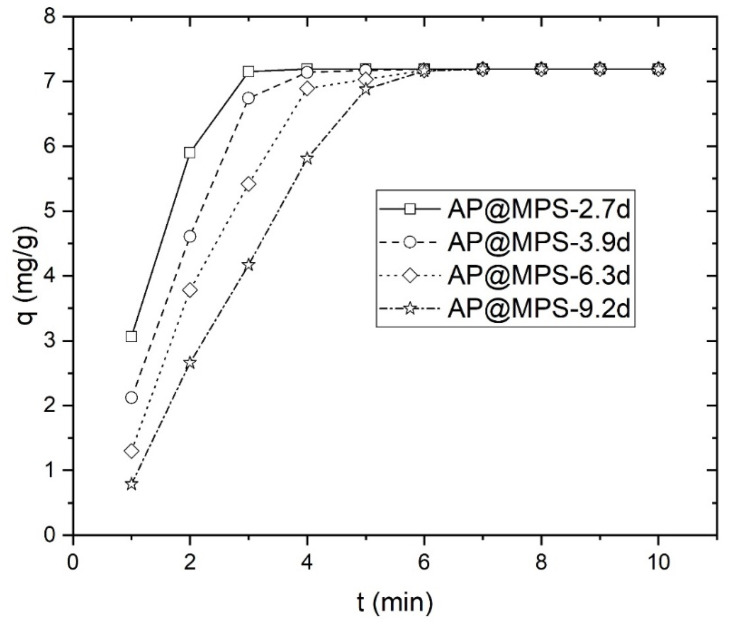
Equilibrium time experiment, the solution pH is 6.0, the initial U(VI) concentration is 3.6 mg·L^−1^, and the weight of the adsorbent is 0.5 g·L^−1^.

The attainment of equilibrium during adsorption is evident within a few minutes, as depicted in Figure 8. Therefore, this study selected a contact time of 30 min for subsequent experiments. While adsorption is theoretically a rapid reaction, achieving equilibrium swiftly, it is noteworthy that Qiu et al. and Gupta et al. have highlighted the limitations of adsorption kinetics in accurately reflecting the actual adsorption process and providing meaningful insights into the adsorption mechanism [19,20]. Furthermore, the pseudo-first-order model’s reliability poses challenges, with notable disparities between the experimental and computed equilibrium adsorption capacity values. Hence, this study emphasizes the equilibrium aspects of U(VI) adsorption rather than its kinetics.

#### 2.2.2. Total Pore Volume vs. U(VI) Capacity

**Figure 9:**
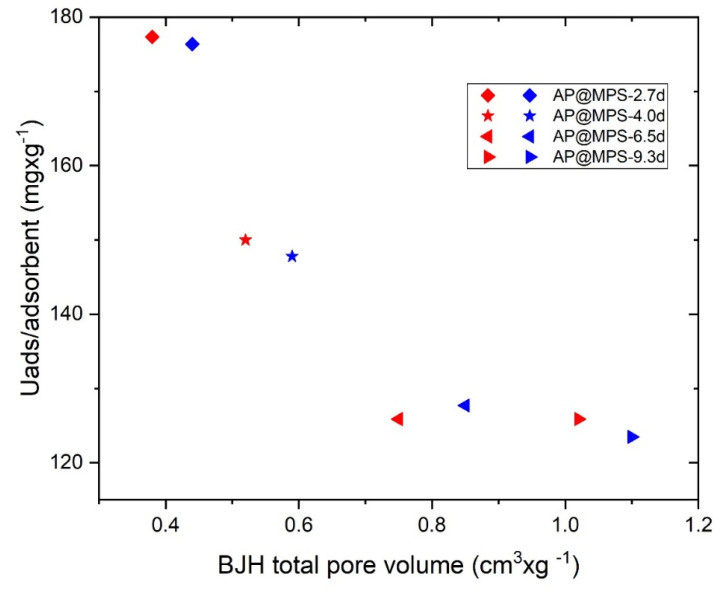
The results of the pore volume experiment under the following conditions: the solution pH is 6.5, the initial U(VI) concentration is 400 mg·L^−1^, the weight of the adsorbent is 1 g·L^−1^, and the adsorption time is 30 min. The blue and red symbols represent two adsorbents with the same surface parameters except for their pore volume.

As indicated in Figure 9, the total pore volume of AP@MPS appears to have no impact on its U(VI) adsorption capacity. According to Equation (10), AP@MPS adsorbed a maximum of 0.31 g∙g^−1^ of U(VI), which would only occupy 0.054 cm^3^∙g^−1^ of the total pore volume. Given this result, it can be concluded that the total pore volume of AP@MPS is a parameter of negligible significance.

### 2.3. Adsorption Modeling, Average Pore Size, and Functional Groups

**Figure 10:**
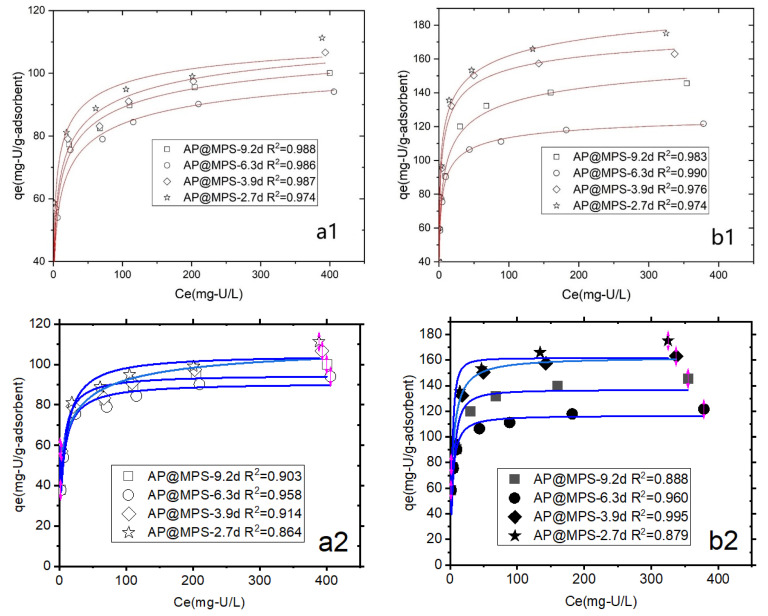
The Langmuir isotherm (number 1, or the two images on top) and the Freundlich isotherm (number 2, or the two images on bottom) of U(VI) onto AP@MPS under different experimental conditions: (**a1**,**a2**) pH = 4.5, (**b1**,**b2**) pH = 6.5. The initial U(VI) concentration ranges from 5 mg·L^−1^ to 500 mg·L^−1^, the weight of the adsorbent is 1 g·L^−1^, and the adsorption time is 30 min.

The adsorption isotherm of AP@MPS is presented in Figure 10, revealing better conformity to the Langmuir isotherm (R^2^ > 0.974) than the Freundlich isotherm (R^2^ > 0.864). Despite the assumption of a heterogeneous distribution of surface energy with the Freundlich adsorption isotherm, the R^2^ value is approximately 0.90, slightly lower than that of the Langmuir adsorption isotherm. While the Langmuir model assumes a monolayer coverage of adsorbates on the surface sites of a homogeneous nature, this study identifies the presence of two distinct types of functional groups on the AP@MPS, showcasing different surface distributions and adsorption energies that impact the goodness of fit to the Langmuir model. Consequently, the R^2^ value derived from the Langmuir adsorption isotherm falls slightly below the ideal value [21,22,23].

Table 3 indicates that AP@MPS exhibits a higher U(VI) adsorption capacity at a pH = 6.5 compared to a pH = 4.5. Among the four adsorbents, AP@MPS-2.7d demonstrates the highest U(VI) adsorption capacity, followed by AP@MPS-3.9d, AP@MPS-9.2d, and AP@MPS-6.3d.

**Figure 11:**
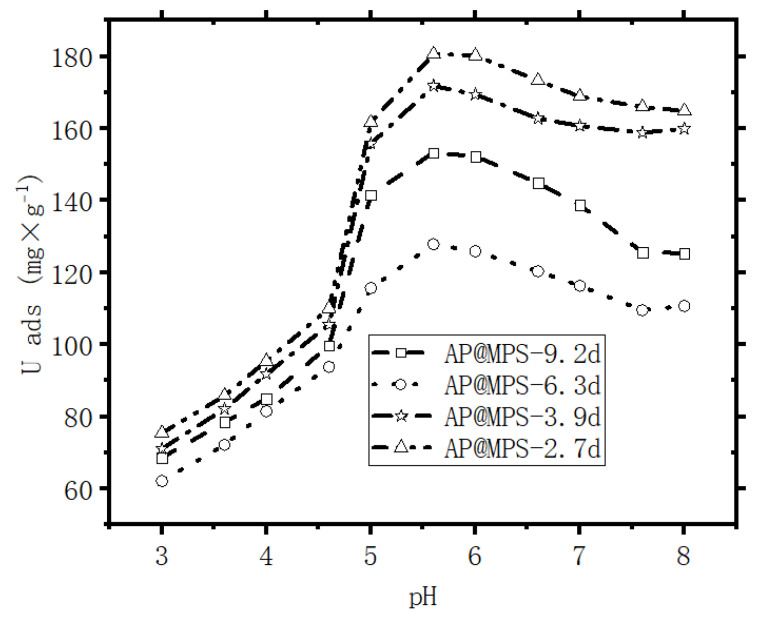
Maximum U(VI) adsorbance capacity experiment; conditions: each point of adsorption performs at a constant pH, the initial U(VI) concentration is 500 mg·L^−1^, the weight of the adsorbent is 1 g·L^−1^, and the adsorption time is 30 min. Adsorption performed at constant pH.

Table 3 provides detailed information on the relationship between the activating site density and the corresponding amount of adsorbed U(VI) ({X^+^}: U^ads^ _Max_), while Figure 11 visually illustrates this connection. Noteworthy is the significant impact of the pore size of AP@MPS on its maximum adsorption capacity. In line with the findings of Kerisit et al. (2013) and Antonchenko (2008), the hydrated U(VI) ion has a radius ranging from 1 to 1.2 nm [24,25]. Based on this information, it can be inferred that, especially with an AP@MPS pore size of 2.7d, U(VI) ions may have more difficulty escaping from the pores. 

### 2.4. PMF and SCFM

**Figure 12:**
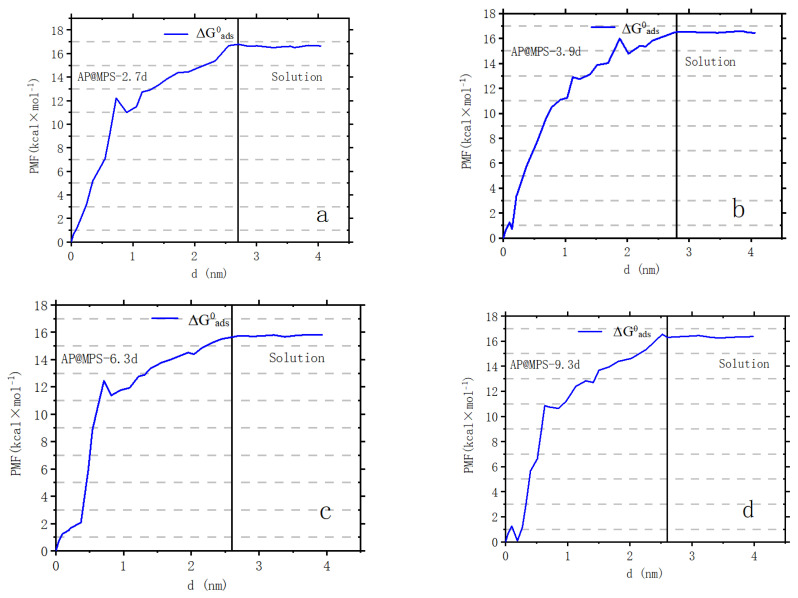
The potential of [U(VI)] ions moving out of AP@MPSs. Panels (**a**–**d**) correspond to AP@MPS-2.7d, AP@MPS-3.9d, AP@MPS-6.3d, and AP@MPS-9.3d, respectively. The black line demarcates the interface between the adsorbent and the solution. The solution pH is 6.5, the initial U(VI) concentration is 500 mg·L^−1^, the weight of the adsorbent is 1 g·L^−1^, and the adsorption time is 30 min.

**Figure 13:**
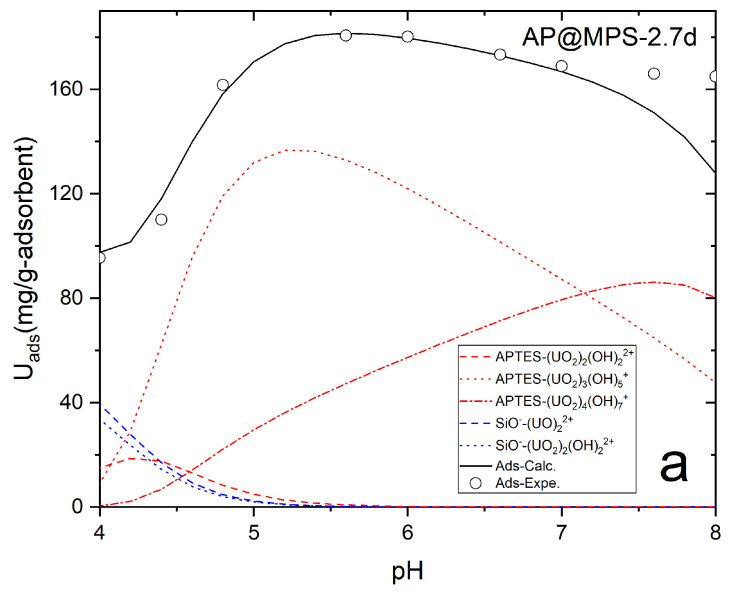

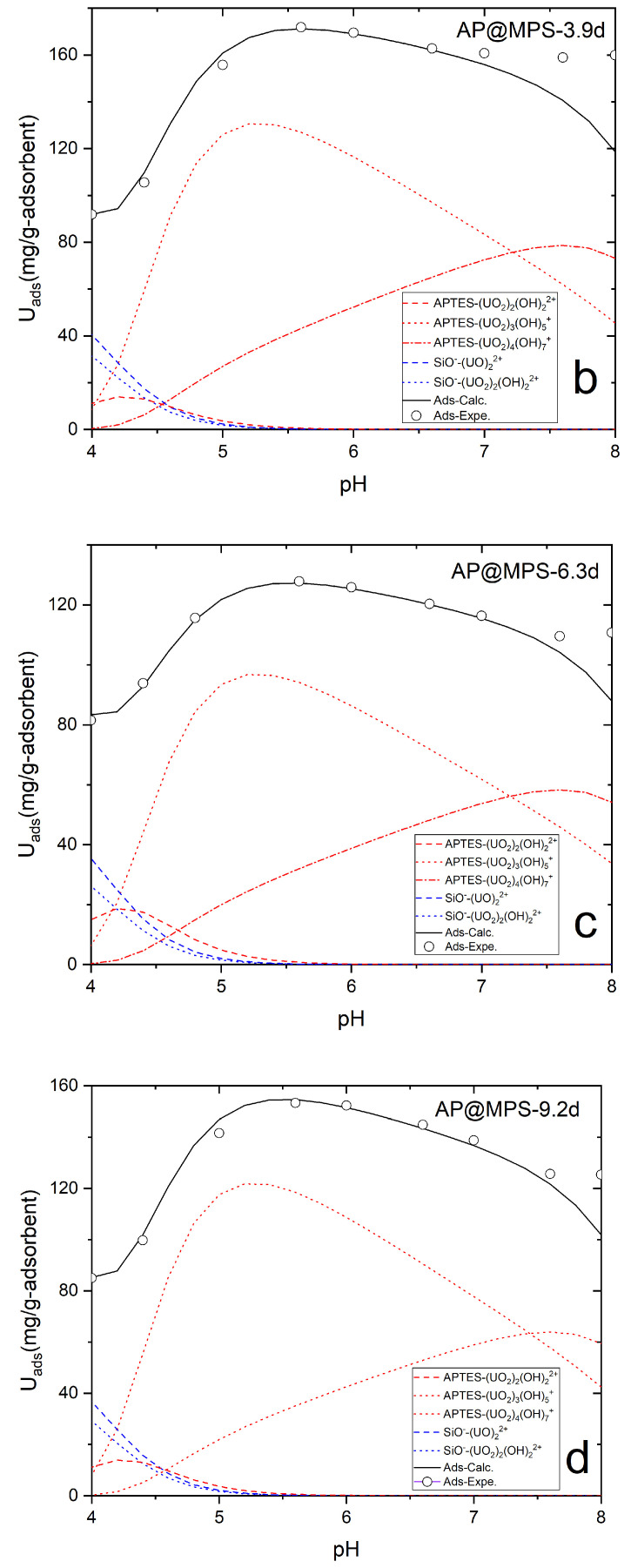
The SCFM results for AP@MPS. Panels (**a**–**d**) represent AP@MPS-2.7d, AP@MPS-3.9d, AP@MPS-6.3d, and AP@MPS-9.3d, respectively. The initiation U(VI) concentration is 500 mg·L^−1^, the weight of the adsorbent is 1 g·L^−1^, and the adsorption time is 30 min.

The calculations for the PMF and SCFM are illustrated in Figure 12 and Figure 13.

**Figure 14:**
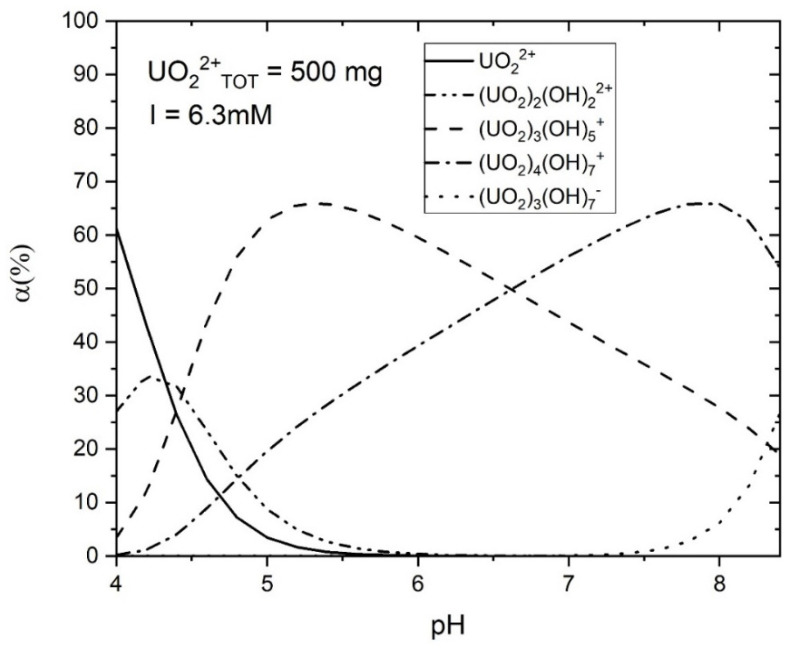
Speciation diagram of aqueous uranyl; initial U(VI) concentration is 500 mg·L^−1^, ionic strength is 6.3 mM.

The calculation results indicate that in an aqueous environment, the U(VI) ions and hydroxyls form complex aqueous U(VI) compounds, with the size of these compounds increasing at higher solution pH levels (Table 4, Figure 14). Figure 14 illustrates U(VI) speciation diagrams under different ionic strengths. Within the experimental solution at pH levels spanning from 4 to 8, two primary types of hydrated U(VI) ions are discernible. Consequently, leveraging the functional group density acquired on the adsorbent surface, the formulation of the surface adsorption reaction can be deduced:

AP@MPS:

(1) $$\equiv {NH}_{4}^{+}-{OH}^{-}+{\left(UO_{2}\right)}_{3}{\left(OH\right)}_{5}^{+}⇌\equiv {NH}_{4}^{+}-{OH}^{-}-{\left(UO_{2}\right)}_{3}{\left(OH\right)}_{5}^{+};K_{1,AP}^{s}$$

(2) $$K_{1,AP}^{s}=\frac{\left\{{(NH}_{4}^{+}\cdots {OH}^{-}){\left({UO}_{2}\right)}_{3}{\left(OH\right)}_{4}^{+}\right\}}{\left\{{NH}_{4}^{+}\right\}[{\left({UO}_{2}\right)}_{3}{\left(OH\right)}_{5}^{+}]}$$

(3) $$\equiv {NH}_{4}^{+}-{OH}^{-}+{\left(UO_{2}\right)}_{4}{\left(OH\right)}_{7}^{+}⇌\equiv {NH}_{4}^{+}-{OH}^{-}-{\left(UO_{2}\right)}_{4}{\left(OH\right)}_{7}^{+};K_{2,AP}^{s}$$

(4) $$K_{2,AP}^{s}=\frac{\left\{{(NH}_{4}^{+}\cdots {OH}^{-}){\left({UO}_{2}\right)}_{4}{\left(OH\right)}_{6}^{+}\right\}}{\left\{{NH}_{4}^{+}\right\}[{\left({UO}_{2}\right)}_{4}{\left(OH\right)}_{7}^{+}]}$$

Table 5 presents the intrinsic stability constants of the UO_2_^2+^ complex, and the adsorption energy is analyzed by dissecting it into specific components: the chemical energy (ΔG^0^_chem_), coulombic force (ΔG^0^_coul_), solvation energy (ΔG^0^_solv_), and lateral interaction (ΔG^0^_lat_). The formula utilized for this analysis is as follows:

(5) $$∆G_{ads}^{0}=∆G_{coul}^{0}+∆G_{chem}^{0}+∆G_{solv}^{0}+∆G_{lat}^{0}$$

For example, James and Healy studied SiO_2_ adsorbing Fe^3+^, Cr^3+^, Co^2+^, and Ca^2+^ [21]; Park and Huang investigated hydrous CdS(s) adsorbing Ni^2+^, Co^2+^, and Zn^2+^ [22]; and Weng et al. explored Cr^6+^ adsorption onto hydrous concrete particles [23]. Additionally, the calculated ΔG^0^_ads_ values align well with the results from the PMF test (Figure 12). It is important to note that the PMF outcomes were used solely for comparison with the calculated results of this study and were not further analyzed. Table 4 displays the ΔG^0^_ads_, ΔG^0^_chem_, ΔG^0^_coul_, ΔG^0^_solv_, and ΔG^0^_lat_ values of AP@MPS-9.2d, while the SCFM results are depicted in Figure 13.

### 2.5. The Mechanism of AP@MPS Adsorbing U(VI) 

From the presented results, it can be inferred that the pH of the solution significantly influences the U(VI) absorption capacity of AP@MPS. When the pH of the U(VI) solution is below 4, the dominant species is UO_2_^2+^, which can bind to the SiO^−^ groups on the surface of the AP@MPS. As the pH increases, OH^−^ ions start to compete for binding sites on the surface, leading to the elution of U(VI) from the AP@MPS and the formation of hydrated U(VI) species (Table 4 and Figure 14). The hydrated U(VI) forms atomic clusters, with positively charged UO_2_^2+^ surrounded by negatively charged OH^−^ ions [24,25,26]. This hydrated U(VI) species can then interact with the NH^4+^ groups on the AP@MPS surface, primarily according to coulombic interactions (ΔG^0^_coul_) (Table 4). As the pH rises beyond 8, the NH^4+^ groups on the AP@MPS surface transform into NH_3_, losing their ability to interact with the hydrated U(VI). Throughout our U(VI) adsorption experiments using AP@MPS, the predominant component of the adsorption energy (ΔG^0^_ads_) in solutions with pHs of 3 to 8 was the coulombic energy (ΔG^0^_coul_). Consequently, AP@MPS exhibits a weak selectivity toward hydrated U(VI).

## 3. Materials and Methods

### 3.1. Chemicals

The tetraethylorthosilicate (TEOS) (98%), (3-aminopropyl)triethoxysilane (APTES) (99%), toluene (99%), and anhydrous ethanol (99.7%) were sourced from Acros, Morris Plains, NJ, USA. The urea (99%), hydrochloric acid (36.5–38.0%), nitric acid (68–70%), aqueous ammonia (25–28%), potassium hydroxide (≥99%), sodium hydroxide (≥99%), and potassium perchlorate (≥99%) were acquired from Fisher Scientific Co., Fair Lawn, NJ, USA. The formaldehyde solution (~37.0%) was obtained from J.T.Baker, Radnor, PA, USA. The uranyl nitrate hexahydrate (UO_2_(NO_3_)_2_∙6H_2_O) (≥99%) was ordered through EHS, the University of Delaware, with the SPI from West Chester, PA, USA, as the distributor.

### 3.2. Synthesis of MPS and AP@MPS

#### 3.2.1. MPS Synthesis 

The mesoporous silica synthesis method utilized in this study was adapted from the work of Hao et al. [17]. Under ambient conditions and vigorous stirring, combine 22.5 mL of anhydrous ethanol with 20 mL of TEOS, followed by 75 mL of deionized water. Adjust the pH to 2 using diluted hydrochloric acid (1 M) and diluted ammonium hydroxide (1 M). Stir the mixture for 4 h. Subsequently, re-adjust the pH of the mixture to 2, dissolve 2.1 g of urea, and add 2.63 mL of formaldehyde under vigorous stirring. Place the mixture in a 50 °C water bath and let it sit for two days. Afterward, filter the intermediate solution using filter paper, collecting the residue, which is porous silica with its template. To remove the template, subject this composite to heat treatment in an oven: 70 °C for 24 h, 200 °C for 2 h, 280 °C for 2 h, and 580 °C for 4 h. The resultant product is porous silica.

#### 3.2.2. AP@MPS Synthesis

**Figure 15:**
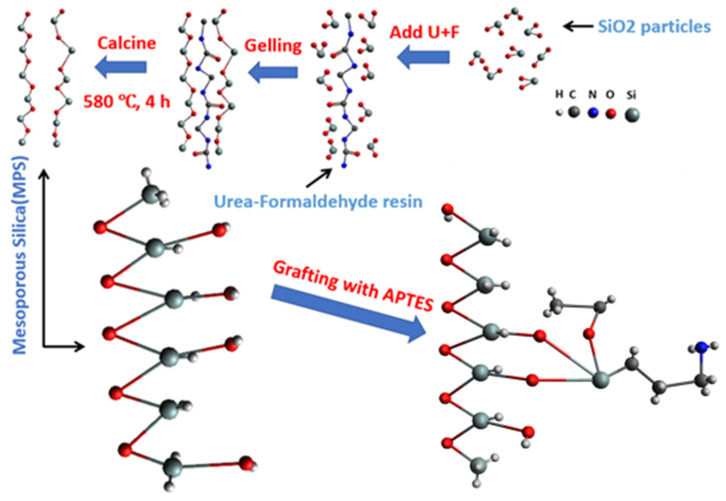
A schematic diagram of the entire synthesis-grafting process of AP@MPS.

Under a nitrogen shield, 1 g of porous silica was placed into a reactor. Subsequently, 100 mL of toluene (99%) and 1 mL of APTES were sequentially added to the reactor. The solution in the beaker was then agitated and heated at 70 °C for 12 h. The solution was filtered after the reaction, and the resulting residue was collected. The residue was subsequently washed with anhydrous ethanol and then dried at 100 °C for 12 h. This process led to the formation of (3-aminopropyl)triethoxysilane-functionalized porous silica (AP@MPS), which was exhibited as a yellow powder. Figure 15 is at representation of the grafting reaction.

### 3.3. Surface Properties

#### 3.3.1. Images and Element Compositions of Samples 

Images of the adsorbents at varying scales were captured using two scanning electron microscopes (SEM). The first one, a JEM-2010F from JEOL Ltd., Tokyo, Japan, was employed to analyze the particle shape of the samples. The second microscope, a ZEISS Auriga 60 CrossBeam from Carl Zeiss Microscopy, White Plains, NY, USA, was utilized to explore the finer details on the adsorbents’ surface. For in-depth analysis, X-ray photoelectron spectroscopy (XPS) was conducted using an ESCALAB 250Xi from Thermo Scientific, Waltham, MA, USA. Operating at 150 W using an Al Kα X-ray source, this technique was employed to investigate the elemental composition on the surface of the samples. Furthermore, an Avance III 400 MHz CP/MAS ^13^C nuclear magnetic resonance (NMR) instrument was employed to analyze the adsorbents’ molecular structure.

#### 3.3.2. Brunauer−Emmett−Teller (BET) and Barrett−Joyner−Halenda (BJH) Analysis

The porous properties of the adsorbents’ surface were evaluated using a BET analyzer, specifically the model ASAP 2020 from Micromeritics, Norcross, GA, USA. The experimental conditions involved N_2_ adsorption/desorption at 77 K. By employing the BET method, both the surface area and pore parameters of the adsorbent were quantified. The pore diameter of the adsorbents was determined using the BJH method. The total pore volume of the adsorbents was computed at a P/P_0_ value of 0.98 via the N_2_ adsorption isotherm. For each zeta potential test, an adsorbent sample with a weight of approximately 0.15 g was utilized. Before the test, this sample was subjected to freeze-drying for a duration of 24 h.

#### 3.3.3. The electrical Properties of Samples

To investigate the surface electrical properties of the adsorbents, a Zetasizer Nano ZS model from Malvern, Westborough, MA, USA, was employed as a particle size analyzer. The sample preparation for the zeta potential test involved two crucial steps: First, the adsorbent was ground and subsequently sieved using a 1200 mesh sieve. Next, the sieved material was placed in a KClO_4_ electrolyte solution for the zeta potential test. The ionic strength of the KClO_4_ electrolyte solution was individually adjusted to three concentrations ranging from 10^−1^ M to 10^−2^ M. 

The point of zero charge (pH_PZC_) can be calculated using the following equation:

(6) $${pH}_{PZC}=\frac{{(pK}_{a1}^{int}+{pK}_{a2}^{int})}{2}$$

The molecular structure of the functional groups on the adsorbent’s surface (Figure 15) provides insight into the anticipated reactions when there is a shift in the solution’s pH. As the pH of the solution undergoes variations, the following reactions are expected to take place:

(7) $$S_{T}=\left\{R-NH_{4}^{+}\right\}+\{R-NH_{3}\}+\left\{Si-OH\right\}+\{{Si-O}^{-}\}$$

(8) $$\left\{R-NH_{4}^{+}\right\}=\{R-NH_{3}\}+\{H^{+}\};K_{a1}^{int}$$

(9) $$\left\{Si-OH\right\}=\left\{{Si-O}^{-}\right\}+\{H^{+}\};K_{a2}^{int}$$

Consequently, utilizing the data gathered from the BET test and the zeta potential test, it becomes possible to compute the total NH^4+^ site density and the total SiO^−^ site density of a given sample. The Supplementary Information contains detailed calculations for the surface acidity, with the corresponding results in Table 6.

### 3.4. Experimental Conditions 

#### 3.4.1. U(VI) Solution Preparation

To prepare the U(VI) solution, a mixture of 10.55 g of uranyl nitrate hexahydrate and 100 mL of nitric acid at 0.2 mol·L^−1^ was introduced into a 1 L volumetric flask. After adding deionized water, the flask was sealed and allowed to sit for a week. After this incubation, the volumetric flask was unsealed and topped with deionized water to reach the mark. The above steps resulted in the initial solution with a total uranium concentration of 5 g·L^−1^. The rest of the experiments in this study involved the preparation of the experimental solutions by diluting this initial solution. For instance, to create a solution with a total uranium concentration of 50 mg·L^−1^, 100 mL of the initial solution was transferred into a 1 L volumetric flask and then diluted with deionized water to the mark.

#### 3.4.2. Analysis of [U(VI)]

The quantification of [U(VI)] in this study employed ICP-MS (Inductively Coupled Plasma with a Mass Selective Detector, model 7500c), manufactured by Agilent Technologies, Santa Clara, CA, USA. This work consistently adjusted the solution’s pH to a specific value throughout the adsorption experiments. The pH adjustments were made using diluted HNO_3_ (1 M) or NaOH (1 M), administered with precision using micro syringes. A water bath was also employed to ensure that the experimental temperature remained consistent at 25 °C.

The subsequent equations (Equations (10) and (11)) were utilized to calculate the adsorption capacity and the percentage of [U(VI)] removal for a given adsorbent:

(10) $$q_{e}=\frac{(C_{0}-C_{ea})V_{a}}{m}$$

(11) $$Removal\;percent\left(\%\right)=\frac{\left(C_{0}-C_{ea}\right)}{C_{0}}\times 100\%$$

In the given equation, *q_e_* is expressed in mg·g^−1^; *m* (g) represents the weight of AP@MPS; and *V_a_* denotes the solution volume (L). The initial uranyl concentration is *C*_0_, and the uranyl concentration at equilibrium is *C_ea_*, expressed in mg·L^−1^.

#### 3.4.3. Duration of Reaching Equilibrium 

The duration required to reach equilibrium holds significant importance as it enables an assessment of whether mass transfer is the rate-limiting step. Mass transfer is likely not the limiting factor if the equilibrium duration is relatively short. The experimental conditions in this section were as follows: a constant solution pH of 6.0, the total uranium concentration set to 3.6 mg∙L^−1^, and the weight of the adsorbent set to 0.5 g∙L^−1^. In a typical experimental setup, the adsorbent was introduced into an agitated reactor containing the U(VI) solution. The solution was filtered once the designated contact time was reached, and the residual [U(VI)] within the filtrate was measured.

#### 3.4.4. The Total Pore Volume of the Adsorbents vs. the Volume of U(VI) Adsorbed

There are four surface properties that could potentially influence the maximum U(VI) capacity of the adsorbent. These properties are the functional group density, average pore size, specific surface area, and total pore volume. Notably, based on a parallel study conducted by the authors, the total pore volume was found to have no impact on the adsorbent capacity [18]. Furthermore, the adsorbents tested in this study demonstrated consistent adsorption behavior.

#### 3.4.5. The Average Pore Size of the Adsorbents vs. the Mass of U(VI) Adsorbed

Drawing from a parallel study, it has been established that the maximum U(VI) capacity is notably influenced by the average pore size of the adsorbent [18]. In this section, the objective is to evaluate the degree of influence exerted by the average pore size of AP@MPS on its maximum U(VI) capacity. Four different AP@MPS samples were prepared to achieve this, each possessing distinct average pore sizes (as detailed in Table 3). The adsorption experiments involving these prepared samples were conducted under consistent conditions: a pH range maintained between 4.0 and 7.0, the initial uranium concentration being 500 mg·L^−1^, the weight of the adsorbent being 1 g·L^−1^, and the contact time being 30 min.

#### 3.4.6. Surface Electrical Properties

Several physics methods, including XEDS, XPS, and NMR, can offer information about the surface element density of an adsorbent. However, while active group atoms may exist on the adsorbent’s surface, it is not assured that all of these atoms will contribute to the surface electrical properties of the adsorbent in a solution. Also, there were challenges in distinguishing activated silanol from other signals and determining whether nitrogen signals indicated activated amino groups; external factors like air exposure may influence these analyses. Furthermore, potential reactions between positively charged amino groups and negatively charged silanol groups could interact, further complicating the situation. Therefore, obtaining group density data for AP@MPS using zeta potential testing in an aqueous solution would provide more substantial evidence. This underscores the significance of the zeta potential properties of adsorbents. Figure 5 illustrates the zeta potential properties of the adsorbents. The experiment employed KClO_4_ as the electrolyte in the solution, with the solution’s ionic strength ranging from 10^−1^ M to 10^−2^ M. Each data point in Figure 5 represents the average result of 30 zeta potential tests at a specific pH value. 

### 3.5. Adsorption Experiments

To ensure accurate adsorption data, the pH of the solution needs to be stable. Thus, two micro syringes were prepared, filled with 1 M nitric acid and 1 M sodium hydroxide, respectively. These syringes were used to adjust and stabilize the solution’s pH. The ionic strength of the solution was below 6.3 mM.

### Langmuir and Freundlich Thermodynamic Isotherms

Both the Langmuir and Freundlich thermodynamic isotherms are robust tools for analyzing adsorption behavior. The Langmuir isotherm operates assuming that homogeneous particles are adsorbed onto a uniform surface. This model posits a monolayer adsorption assumption, with no interactions between the adsorption sites. The Freundlich isotherm, on the other hand, is an empirical model that suggests a relationship between the concentration of the adsorbate in the solution and the maximum adsorption capacity of the adsorbent.

The mathematical expression of these two isotherms is as follows:

(12) $$Langmuir\;isotherm:\;q_{e}=\frac{q_{max}C_{e}K_{L}}{1+C_{e}K_{L}}$$

(13) $$Freundlich\;isotherm:\;q_{e}=K_{f}C_{e}^{\frac{1}{n}}$$

where *q_max_* represents the maximum capacity of the U(VI) adsorption in g/g, *C_e_* signifies the equilibrium concentration of U(VI) in g/L. *K_L_* and *K_f_* stand for the Langmuir constant and Freundlich constant, respectively. 

Previous studies have indicated that the maximum capacity of AP@MPS is achieved with a solution pH ranging from 4 to 8. Thus, two pH points, 4.5 and 6.5, were selected for the adsorption experiments. The initial uranium concentration range spanned from 5 mg/L to 500 mg/L, with an adsorbent weight of 1 g/L and a contact time of 30 min.

### 3.6. The Speciation Diagram of [U(VI)]

This study adopted the MINTEQ software v3.1 and the Eidgenössische Technische Hochschule (ETH) database to simulate the speciation of [U(VI)] under a specific ionic strength. Essential details such as the solute concentrations (U(VI) and NO^3−^) and ionic strength can be entered into the primary interface of the software (“Temperature” set as 25 °C, “Concentration unit” set as Molal). Moving to the multi-problem/Sweep page, we selected the “Sweep: one parameter is varied” option. pH was chosen as the variable parameter, starting from a value of 2. We configured the “state the number of problems” option to 80, incorporating an increment of 0.1 between values. Following these settings, we executed the process by clicking the “save and back” button and selecting “run” on the software’s main interface. This sequence initiates the generation of the percentage distribution of components at each pH step. These results could be copied and transferred into software such as Excel to construct an aqueous U(VI) speciation diagram.

### 3.7. The PMF Analysis

Muegge and Martin pioneered applying the Potential of Mean Force (PMF) model to predicting the binding affinity in protein–ligand complexes based on their 3D structure [27,28]. Extending this paradigm, Kovalenko and Hirata expanded the model to compute the orientationally dependent Potential of Mean Force (PMF) between polyatomic solutes immersed in a polar molecular solvent [29]. Further refinement by Muegge introduced general metal ion atom types (ME), facilitating interactions with ions like Zn, Ca, K, Mg, Mn, and Fe. This enhancement allowed the estimation of binding affinities involving nitrogen atoms and ME [30]. Trzesniak et al. proposed a PMF-based method for computing the Gibbs free energies in different states in 2007 [31]. Nalaparaju and Jiang employed the PMF to simulate the exchange of Pb^2+^ ions with Na^+^ ions in rho-zeolite-like MOF, uncovering the more potent bond energy of Pb^2+^ ions to the MOF [32]. Investigating the bond energy between amino-derived MOF and uranium, Li et al. reported the uranium adsorption energy (ΔG^0^_ads_) on the MOF without elaborating on the ΔG^0^_ads_ composition [13].

Independent umbrella sampling was conducted for each adsorbent, consisting of 30 snapshots, each lasting 1 nano-second. The sampling window dimensions were set at 0.5 nm × 0.5 nm, with a harmonic biasing force constant of 10 kJ/mol·A^2^. Subsequently, the estimation of the Potential of Mean Force (PMF) for the adsorbents was carried out using the Weighted Histogram Analysis Method (WHAM) [33,34].

## 4. Comparison and Discussion

### 4.1. The Beta Study 

In 2016, the author initiated a beta study [11,26] to assess the efficacy of amino-functionalized MPS in removing aqueous U(VI). The three adsorbents synthesized during the beta study demonstrated the effective removal of aqueous U(VI). The results indicated that, for these adsorbents, a higher pH_PZC_ was associated with a larger U(VI) capacity. In an actual wastewater U(VI) removal experiment, AFPSs showed noticeable selectivity toward U(VI), although the specific removal mechanism remained unclear.

Additionally, the beta study investigated the U(VI) desorption from loaded adsorbents and subsequent adsorbent regeneration. The findings suggested that 0.1 mol × L^−1^ HNO_3_ could rapidly elute the adsorbed U(VI). After 5 and 15 adsorption-desorption cycles, the adsorbents retained 60–70% and 50–60% of their maximum capacity, respectively.

### 4.2. The Parallel Study 

The authors have conducted a series of studies in recent years to analyze the mechanism of adsorption of U(VI) in AFPS further. For instance, in a parallel study, they assessed the distribution of the adsorption energy (ΔG^0^_ads_) in AEPTES-functionalized porous silica with multiple average pore sizes [18]. According to this investigation, the adsorption curve aligns well with the predictions of the SCFM. The U(VI) adsorption energy of AE@MPS-9.3d, as calculated using the SCFM, reveals that at a pH < 4, ΔG^0^_ads_ is primarily influenced by the chemical energy (ΔG^0^_chem_) between the SiO^−^ and UO_2_^2+^. In the pH range of 4 to 8, with an increase in the OH^−^ concentration, the SiO^−^ attaching to UO_2_^2+^ is replaced by OH^−^, resulting in ΔG^0^_ads_ being the combined effect of the ΔG^0^_chem_ and ΔG^0^_coul_ between the AFPS and UO_2_^2+^. For AE@MPS, ΔG^0^_chem_ is slightly weaker than ΔG^0^_coul_. Elevating the pH enhances the contribution of ΔG^0^_chem_ to the total adsorption energy.

### 4.3. Discussion 

#### 4.3.1. Insights from the Beta Study

The beta study’s outcomes have shown that AFPS adsorbents effectively eliminate U(VI) from water within a pH range of 3 to 9. Analysis of the thermodynamic isotherms indicates that adsorption is the primary process for U(VI) removal, and the ease with which it can be desorbed using acid treatment further confirms this. The rapid achievement of an adsorption equilibrium suggests that mass transfer is not a rate-limiting step. The regeneration process highlights the durability of AFPS, and its solubility in alkaline conditions offers a practical method for disposal by incorporating used AFPS into concrete for applications in anaerobic environments, such as underground mining.

Although the beta study successfully positioned AFPS as a potential U(VI) adsorbent, it used adsorbents with a consistent pore size of about 8 nm, leaving room for investigating how different pore sizes might affect the adsorption efficiency. Thus, it is essential to create MPSs with adjustable pore sizes to assess the effect of synthesis parameters on the surface characteristics.

Furthermore, the beta study’s use of XPS and XEDS to analyze the functional group density on the AFPS surfaces has limitations, as these techniques can only detect the silica signal and cannot accurately measure the silanol group density on the AFPS. While physical methods can be used to measure the density of amino groups, the activation state of these groups during adsorption remains uncertain. The thermodynamic isotherms and pseudo-first-order kinetic models applied in the beta study provide only partial insights into the adsorption process, and a more comprehensive understanding still needs to be provided.

To further unveil the underlying mechanisms of adsorption, the parallel and present studies have synthesized AFPS with varying average pore sizes to adsorb U(VI) from water. The surface group densities of these adsorbents were determined using zeta potential measurements, and the SCFM was used to evaluate the distribution of the adsorption-free energy.

#### 4.3.2. Insights from the Parallel Study

The parallel research [18] has elucidated the adsorption process of U(VI) using AE@MPS, implying that a similar mechanism may apply to AP@MPS in the current study. Given the critical influence of the pore size on AE@MPS’s maximum U(VI) adsorption capacity, it is expected that the ideal pore size for AP@MPS would be distinct. This work also seeks to contrast its findings with the parallel study to elucidate any disparities in the adsorption energy distribution, which could arise from the differences in the functional group geometry and dimensions between AE@MPS and AP@MPS.

#### 4.3.3. Findings from the Current Study 

Table 7 showcases recent discoveries pertinent to this research, which provides novel perspectives while employing methodologies and materials similar to a prior study by the same authors [18]. The surface amino group ratio enhancement resulted in a higher pH_PZC_. Additionally, AP@MPS, characterized by an average pore size of 2.7 nm, demonstrated a superior X^+^:U^ads^ _Max_ (as detailed in Table 3). The ΔG^0^_ads_ values derived from the experimental data were found to be in concordance with those from PMF simulations. The SCFM analysis across a pH range of 4 to 8 underscored the critical role of pH in the adsorption of U(VI) by AP@MPS. Minimal U(VI) adsorption by AP@MPS was noted at pH levels below 2, with U(VI) primarily interacting with SiO^−^ between pHs of 2 and 4 and with NH^4+^ at pHs from 4 to 8. Figure 14 illustrates that U(VI) species carry a positive charge within the pH range of 4 to 8, corresponding to the amino groups’ positive charge. The attraction between the positively charged amino groups and the hydrated uranyl is facilitated by the uneven distribution of the OH- groups within the hydrated uranyl particles. The substantial size of uranyl (0.62–0.64 nm) and the consequent larger size of hydrated uranyl (1.2–1.4 nm) enable the smaller OH- groups (0.096–0.098 nm) to reposition, creating an uneven charge distribution that allows the positively charged amino groups to attract the net-positively charged hydrated uranyl through its evenly distributed hydroxyls.

The referenced study [18] and the current research suggest that an elevation in the surface amino group ratio elevates the pH_PZC_ of AFPS and the maximum U(VI) adsorption capacity. The large size and strong electropositivity of the hydrated U(VI) cations result in the repulsion of other aqueous cations. The positively charged amino groups on the adsorbent surface cannot adsorb these cations directly, leading to a selectivity bias against U(VI) ions.

A comparative analysis of the SCFM results reveals notable disparities in the ΔG^0^_chem_ and ΔG^0^_coul_ values between the adsorbent from the previous study and AP@MPS. In the former, both energy components contribute equally to ΔG^0^_ads_, whereas in AP@MPS, the ΔG^0^_coul_ values are considerably larger, playing a more significant role in ΔG^0^_ads_. Upon examining the functional group structure of the adsorbents from the previous study, an increase in the number of amino groups per active site could lead to a higher proportion of ΔG^0^_chem_, potentially enhancing the U(VI) adsorption capacity and selectivity over other ions. On the other hand, a reduction in the number of amino groups per functional group, as observed in AP@MPS, would increase the proportion of ΔG^0^_coul_, facilitating the elution of the adsorbed U(VI).

## 5. Conclusions

This study underscores the pivotal influence of the surface group density, average pore size, and solution pH on the maximum U(VI) adsorption capacity of AP@MPS. These findings offer valuable insights for formulating similar U(VI) removal adsorbents. Achieving the optimal results may involve controlling the average pore size of the AP@MPS during synthesis to align with the dimensions of the hydrated U(VI) in the target solution. As determined using the SCFM, the adsorption energy distribution elucidates the mechanism by which AP@MPS adsorbs U(VI). This knowledge proves crucial for designing adsorbents with amino functional groups to remove aqueous U(VI) effectively. However, it is essential to acknowledge the limitations of the SCFM, particularly in scenarios where high acidity or chemical reactions prevail. The assumption of no electron transfer or chemical reaction in the solution may introduce an unavoidable inaccuracy into the SCFM under such conditions.

## Figures and Tables

**Table 1:** Acronyms and the substances they represent.

TEOS	Tetraethylorthosilicate
APTES	(3-aminopropyl)trimethoxysilane
AP@MPS	(3-aminopropyl)trimethoxysilane-functionalized porous silica
AP@MPS-2.7d	AP@MPS with average pore size of 2.7 nm and BET area of 380 m^2^·g ^−1^
AP@MPS-3.9d	AP@MPS with average pore size of 3.9 nm and BET area of 340 m^2^·g ^−1^
AP@MPS-6.3d	AP@MPS with average pore size of 6.3 nm and BET area of 170 m^2^·g ^−1^
AP@MPS-9.2d	AP@MPS with average pore size of 9.2 nm and BET area of 240 m^2^·g ^−1^
SCFM	Surface Complex Formation Model
PMF	Potential of Mean Force
WHAM	Weighted Histogram Analysis Method

**Table 2:** Results of BET analysis of adsorbents.

Adsorbent	BET Surface Area (m^2^·g ^−1^)	BJH Average Pore Diameter (nm)
AP@MPS-9.3d	170	9.2
AP@MPS-6.3d	240	6.3
AP@MPS-3.9d	340	3.9
AP@MPS-2.7d	380	2.7

**Table 3:** The relationship between the functional group density of adsorbents and the amount of [U(VI)] adsorbed at pH = 6.5.

Adsorbent	Surface Area m^2^·g^−1^	Pore Diameter nm	{X^−^} mM·L^−^	{X^+^} mM·L^−1^	
AP@MPS					X^+^: U^ads^ _Max_
AP@MPS-9.2d	240	9.2	0.81	1.25	1:0.368
AP@MPS-6.3d	170	6.3	1.01	1.13	1:0.370
AP@MPS-3.9d	340	3.9	0.98	1.26	1:0.436
AP@MPS-2.7d	380	2.7	1.04	1.23	1:0.546

**Table 4:** The adsorption energy of AP@MPS-9.2d in kcal∙mol^−1^.

Equation	ΔG^0^_ads_	ΔG^0^_chem_	ΔG^0^_lat_	ΔG^0^_coul_	ΔG^0^_solv_
APTES+(UO_2_)_3_(OH)_5_^+^	−16.32 ± 0.1	−2.35 ± 0.1	0	−14.21 ± 0.1	0.24 ± 0.1
APTES+(UO_2_)_4_(OH)_7_^+^	−15.58 ± 0.1	−1.61 ± 0.1	0	−14.21 ± 0.1	0.24 ± 0.1
APTES+(UO_2_)_2_(OH)_2_^2+^	−12.10 ± 0.1	16.09 ± 0.1	0	−28.43 ± 0.1	0.24 ± 0.1
SiO^−^ + UO_2_^2+^	−45.53 ± 0.2	−17.34 ± 0.2	0	−28.43 ± 0.2	0.24 ± 0.2
SiO^−^ + (UO_2_)_2_(OH)_2_^2+^	−44.96 ± 0.2	−16.77 ± 0.2	0	−28.43 ± 0.2	0.24 ± 0.2

**Table 5:** The intrinsic stability constants of the UO_2_^2+^ complex.

Adsorbent	Amino Groups			Silica Groups	
	pK^s^_(UO2)3(OH)5+_	pK^s^_(UO2)4(OH)7+_	pK^s^_(UO2)2(OH)22+_	pK^s^_(UO2)2(OH)22+_	pK^s^_UO22+_
AP@MPS-9.2d	−2.86	−2.73	−2.12	−7.98	−7.88
AP@MPS-6.3d	−2.76	−2.69	−2.25	−8.01	−7.88
AP@MPS-3.9d	−2.89	−2.82	−2.12	−8.02	−7.91
AP@MPS-2.7d	−2.91	−2.86	−2.25	−8.02	−7.95

**Table 6:** The surface electrical properties of adsorbents.

Sample	I (M)	σ_N_ (C·m^−2^)	−σ_Si_ (C·m^−2^)	{X_N_^+^} (mM·L^−1^)	{X_Si_^−^} (mM·L^−1^)	pK^int^_N_	pK^int^_si_	pH_PZC_
AP@MPS-2.7d	10^−1^	0.315	0.259	1.24	1.02	6.69	4.95	5.82
	3 × 10^−2^	0.297	0.269	1.17	1.06			
	10^−2^	0.317	0.264	1.25	1.04			
AP@MPS-3.9d	10^−1^	0.352	0.278	1.24	0.98	6.70	4.96	5.83
	3 × 10^−2^	0.355	0.278	1.25	0.98			
	10^−2^	0.358	0.275	1.26	0.97			
AP@MPS-6.3d	10^−1^	0.631	0.580	1.11	1.02	6.63	4.97	5.80
	3 × 10^−2^	0.652	0.571	1.15	1.01			
	10^−2^	0.633	0.591	1.12	1.04			
AP@MPS-9.2d	10^−1^	0.511	0.326	1.27	0.81	6.66	4.94	5.80
	3 × 10^−2^	0.503	0.322	1.25	0.80			
	10^−2^	0.490	0.323	1.22	0.83			

**Table 7:** Recent results related to the present study.

No.	Material	Experiment Conditions	Performance	Ref.
1	AEPTES-functionalized porous silica	pH_0_ = 6.5; [U]_0_ = 600 mg × L^−1^; [X] = 1 g × L^−1^; T = 25 °C	Γ_m_ = 551.97 mg-U/g; R = 92% @ 0.5 h	[18]
2	Phosphorylated hyper-cross-linked polymers	pH_0_ = 7; [U]_0_ = 60 mg × L^−1^; [X] = 0.2 g × L^−1^; T = 25 °C	Γ_m_ = 297.14 mg-U/g; R = 85% @ 0.08 h	[35]
3	Hypercrosslinked phenylalaninol	pH_0_ = 7; [U]_0_ = 100 mg × L^−1^;	Γ_m_ = 369.5 mg-U/g; R = 56.3% @ 2 h	[36]
4	Amidoxime-functionalized Fe_3_O_4_@TiO_2_ microspheres	pH_0_ = 6; [U]_0_ = 23.8 mg × L^−1^; [X] = 0.2 g × L^−1^; T = 25 °C	Γ_m_ = 313.6 mg-U/g; @ 12 h	[37]
5	Amidoximized porous polyacrylonitrile	pH_0_ = 6; [U]_0_ = 117 mg × L^−1^; [X] = 0.1 g × L^−1^; T = 25 °C	Γ_m_ = 1058 mg-U/g; R = 90.50% @ 5 h	[38]
6	Layered silicate RUB-15	pH_0_ = 6; [U]_0_ = 20 mg × L^−1^; [X] = 0.5 g × L^−1^; T = 25 °C	Γ_m_ = 152 mg-U/g; R = 74% @ 12 h	[39]
7	Amide and phosphorous functionalized silica	pH_0_ = 1.7; [U]_0_ = 120 mg × L^−1^; [X] = 5 g × L^−1^	Γ_m_ = 95 mg-U × g_ads_^−1^; R = 40% @ 24 h	[4]
8	Ion-imprinted mesoporous silica	[U]_0_ = 1.68 mM; pH_0_ = 0; [X] = 1 g × L^−1^; T = 25 °C	Γ_m_ = 80 mg-U × g_ads_^−1^; R = 20% @ 0.67 h	[1]
9	Amino-functionalized magnetic titanate nanotubes	pH_0_ = 6; [U]_0_ = 200 mg × L^−1^; [X] = 0.4 g × L^−1^; T = 25 °C	Γ_m_ = 509.89 mg-U × g_ads_^−1^; R = 83% @ 0.83 h	[7]
10	Copper bimetallic central MOFs	pH_0_ = 3; [U]_0_ = 30 mg × L^−1^; T = 25 °C	Γ_m_ = 617.814 mg-U × g_ads_^−1^@ 12 h	[40]
11	Copper/iron bimetallic central MOFs	pH_0_ = 7; [U]_0_ = 60 mg × L^−1^; T = 25 °C	Γ_m_ = 354.724 mg-U × g_ads_^−1^@ 12 h	[40]
12	APTES-functionalized porous silica	pH_0_ = 6.5; [U]_0_ = 600 mg × L^−1^; [X] = 1 g × L^−1^; T = 25 °C	Γ_m_ = 381.44 mg-U/g; R = 63.6 % @ 0.5 h	present work

Γ_m_ = maximum adsorption capacity; pH_0_ = initial pH; [X] adsorbent concentration_;_ [U]_0_ = initial uranium concentration.

## Data Availability

The data presented in this study are available in article and Supplementary Materials.

## Supplementary Materials

The following supporting information can be downloaded at https://www.mdpi.com/article/10.3390/molecules29040803/s1.
