# Supplementary material for: Analyzing (3-Aminopropyl)triethoxysilane-Functionalized Porous Silica for Aqueous Uranium Removal: A Study on the Adsorption Behavior
Source: Molecules. 2024 Feb 9;29(4):803. doi: 10.3390/molecules29040803 (PMC10891806; doi:10.3390/molecules29040803)
Supplement: Supplementary file 1 [file molecules-29-00803-s001.zip › molecules-2847449-supplementary.pdf]

Analyzing (3-Aminopropyl)triethoxysilane-Functionalized Porous Silica for Aqueous Uranium Removal: A Study on the Adsorption Energy

Kegang Wei [1,2,*] and Chin-Pao Huang [1]

[1] Aquatic Chemistry Lab, Civil and Environmental Engineering Department, University of Delaware, Newark, DE 19711, USA; huang@udel.edu
[2] Jiangxi Copper Technology Institute Co., Ltd., Nanchang 33000, China
* Correspondence: kegang@udel.edu

Thickness of the double layers:

In a solution, the solid surface undergoes hydration, leading to the formation of surface hydroxyl groups and exhibiting amphoteric behavior in response to the solution's pH. This phenomenon, also known as surface acidity, holds significant importance when studying surface reactions like

adsorption. When dealing with low metal concentrations in a solution, the interaction between the adsorbate and the adsorbent's functional sites can be thermodynamically modeled. The Surface Complex Formation Model (SCFM), proven successful in describing adsorption mechanisms with high predictability (James and Healy, 1972; Hao and Huang, 1986), can shed light on how amino groups interact with UO_2^{2+} groups. By performing comprehensive calculations using the speciation diagram of [U(VI)] across a pH range, the SCFM can be tailored to refine our understanding of the bonding mechanism between the amino groups and UO_2^{2+} groups. This study provides valuable insights for guiding future experimental investigations. For a dilute aqueous solution with ε_r = 78.54 at 298 K, the diffusion layer thickness can be expressed using the following equation.

$$\kappa^2 = \frac{1000e^2N_A}{\varepsilon_r\varepsilon_0 kT} \times 2C^* \quad \text{(S1)}$$

where

e is the polar molecule ionic charge;

N_A is Avogadro’s number;

ε_0 is the permittivity of empty space;

ε_r is the relative permittivity of water;

C^* is the bulk z_+:z_- electrolyte concentration in mol·L^{-1};

k is the Boltzmann constant;

T is the temperature in Kelvins.

As a simplified equation, we obtain:

$$\kappa = (3.29 \times 10^7)zC^{*1/2} \quad \text{(S2)}$$

κ is given in cm^{-1}.

Thus, the characteristic thickness of the diffuse layer versus C^* can be obtained:

Table S1. The thickness of the diffuse layer

C*(M)	1/κ(nm)
10^{-1}	0.96
3×10^{-2}	1.76
10^{-2}	3.04
10^{-3}	9.62
10^{-4}	30.4

*. For a 1:1 electrolyte at 298 K in water; **. $C^* = n^0/N_A$ where N_A is Avogadro's number

Surface electrical properties calculation:

According to the Gouy–Chapman theory, the relationship between the electrolyte concentration and the reciprocal thickness (κ) of the diffuse electrical double layer is:

$$\kappa = (3.29 \times 10^7) z C^{*1/2} \quad \text{(S3)}$$

where z is the charge of the ion; C^* is the bulk z_+:z_- electrolyte concentration in mol·L^{-1}; and κ is given in cm^{-1}.

The thickness (κ^{-1}) of the diffuse electrical double layer is considered the distance of the shearing plane. Thus, the potential at the surface of the adsorbent (ψ_0) is:

$$\psi_0 = \frac{asinh\left(\frac{\sigma_p}{0.1174 \times c^{1/2}}\right)}{19.46} \quad \text{(S4)}$$

$$\psi = \psi_0 exp\ (-\kappa d) \quad \text{(S5)}$$

where σ_p is the surface density ($C{\cdot}m^{-2}$); ψ_0 is the surface potential (V); d is the distance from the particle surface (nM); ψ is the potential at distance d (V).

James et al. (1982) introduced this method to calculate the surface chemical properties of aqueous colloids. From eq(6-8) in the main text, the surface charge intensity of the adsorbent decreases when the electrolyte concentration increases. When the net charge on the surface of the adsorbent is zero, the corresponding pH value is called pH_{PZC}. From Table 2 and Figure 3, we see the calculated pH_{PZC} well agreed with the experimental data.

Figure 8 elucidates the distribution of the surface acidity groups, namely SiOH-SiO^- and R-NH_3/RNH_4^+ sites, as a function of the pH. The interaction of the curves demonstrates that the surface concentrations of SiO^- and SiOH and R-NH_3 and R-NH_4^+ are similar. At a pH < 1, the functional groups on the surface of the adsorbent are {SiOH} and {NH_4^+}; at 4 < pH < 8, {SiOH} turned into {SiO^-}, while {NH_4^+} remain unchanged; at a pH > 10, the surface electrical properties of the adsorbent are only provided by {SiO^-} groups. Combining the information provided in Figure 3, the net surface potential of AP@MPS is provided by {NH_T} at a pH < 1 and by {$SiOH_T$} at a pH > 10.

Based on previous studies, such as Hair et al. (1970) and Corapcioglu et al. (1987), the density of the functional group can be shown as:

(a) at pH < pH_{pzc},

$$|\sigma_N| = \frac{\{R-NH_4^+\}}{S} \times F \tag{S6}$$

Thus,

$$\{R - NH_4^+\} = \frac{|\sigma_N| \times S}{F} \tag{S7}$$

(b) at pH > pH_{pzc},

$$|\sigma_{Si}| = \frac{\{SiO^-\}}{S} \times F \quad \text{(S8)}$$

$$\{SiO^-\} = \frac{|\sigma_{Si}| \times S}{F} \quad \text{(S9)}$$

The equation for the intrinsic constants of the adsorbents us expressed as:

From eq(S6), at pH > pH_{PZC}, the total positive charge density is equal to $\{NH_T\}$, and the net surface charge is equal to $\{NH_T\}$-$\{\{SiOH_T\}$-$\{SiO^-\}\}$ or $\{NH_T\}$-$\{SiOH_T\}$+$\{SiO^-\}$; thus,

$$K_N^{int} = \frac{\{H^+\}\{NH_3\}}{\{NH_4^+\}} = [H^+] \times P^{-1} \times \frac{\{NH_T\}-\{SiOH_T\}+\{SiO^-\}}{\{SiOH_T\}-\{SiO^-\}}; \ @pH > pH_{zpc} \quad \text{(S10)}$$

From eq(S7), at pH < pH_{PZC}, the total negative charge density is equal to $\{SiOH_T\}$, and the net surface charge is equal to $\{SiOH_T\}$-$\{\{NH_T\}$-$\{NH_4^+\}\}$ or $\{SiOH_T\}$-$\{NH_T\}$+$\{NH_4^+\}$; thus,

$$K_{Si}^{int} = \frac{\{H^+\}\{SiO^-\}}{\{SiOH\}} = [H^+] \times P \times \frac{\{NH_T\}-\{NH_4^+\}}{\{SiOH_T\}-\{NH_T\}+\{NH_4^+\}}; \ @pH < pH_{zpc} \quad \text{(S11)}$$

where NH_T is the total concentration of active amino sites in mol·L^{-1}; $SiOH_T$ is the total concentration of active silanol sites in mol·L^{-1}; $\{R\text{-}NH_4^+\}$ is the surface amino density of AP@MPS in mol·g^{-1}; $\{SiO^-\}$ is the surface silanol density of AP@MPS in mol·g^{-1}; and S is the BET area of the materials in m^2·g^{-1}.

The surface density σ_p ($C\cdot m^{-2}$) and surface potential ψ_0 (V) are related as follows:

$$\sigma_p = (8RT\varepsilon_r\varepsilon_0 c \times 10^3)^{\frac{1}{2}} \times sinh\left(\frac{Z_i F \psi_0}{2RT}\right) \quad \text{(S12)}$$

where

R is the molar gas constant ($R = 8.314\ J\cdot mol^{-1}\cdot K^{-1}$);

T is the temperature in Kelvins (K) ;

ε_0 is the permittivity of empty space ($\varepsilon_0 = 1$);

ε_r is the relative permittivity of water at 25℃ ($\varepsilon_r = 78.54$);

c is the electrolyte concentration (M);

z_i is the electrolyte charge z_+ versus z_-; in this case, $z_i = 1$;

F is the Faraday constant ($F = 96485 C \cdot mol^{-1}$).

At 25℃, eq(S12) can be simplified:

$$\sigma_p = 0.1174 \times c^{\frac{1}{2}} \times sinh(19.46 \times Z_i \times \psi_0) \quad \text{(S13)}$$

We reform eq(S13):

$$\psi_0 = \frac{asinh\left(\frac{\sigma_p}{0.1174 \times c^{1/2}}\right)}{19.46} \quad \text{(S14)}$$

From the Debye–Hückel equation,

$$\psi = \psi_0 exp\,(-\kappa d) \quad \text{(S15)}$$

where

d is the distance from the particle surface (*nM*);

ψ is the potential at distance d (*V*).

When a particle moves in a solution, its surface is wrapped in a tight liquid film. The interface between the liquid film and the bulk solution is called the shear plane (slipping plane), and the distance between the particle surface and the shear plane is d. The electro-properties obtained using zeta potential tests are that of the shear plane (Wiersema et al. 1966, James and Healy 1972).

Thus, assuming $d = \kappa^{-1}$,

$$\zeta \approx \psi = \psi_0 \exp\,(-1) \quad \text{(S16)}$$

ζ is the zeta potential data of particle (*V*).

Therefore, from the results of the zeta potential text and the result from Table S1 ,

(c) At pH < pH_{pzc},

$$|\sigma_N| = \frac{\{R-NH_4^+\}}{S} \times F \quad \text{(S17)}$$

Therefore,

$$\{R - NH_4^+\} = \frac{|\sigma_N| \times S}{F} \quad \text{(S18)}$$

(d) At pH > pH_{pzc},

$$|\sigma_{Si}| = \frac{\{SiO^-\}}{S} \times F \quad \text{(S19)}$$

$$\{SiO^-\} = \frac{|\sigma_{Si}| \times S}{F} \quad \text{(S20)}$$

where

{R-NH₄⁺} is the surface amino density of AP@MPS in mol·g^{-1};

{SiO⁻} is the surface silanol density of AP@MPS in mol·g^{-1};

S is the BET area of the materials in m^2·g^{-1}.

From eq(7-9) in the main text:

$$S_T = \{R - NH_4^+\} + \{R - NH_3\} + \{Si - OH\} + \{Si - O^-\} \quad \text{(S21)}$$

$$\{R - NH_n^+\} = \{R - NH_{n-1}\} + \{H^+\}\,; \quad K_{a1}^{int} \quad \text{(s22)}$$

$$\{Si - OH\} = \{Si - O^-\} + \{H^+\}\,; \quad K_{a2}^{int} \quad \text{(S23)}$$

In the main text, in the **Surface electrical properties** section, the net surface potential of AP@MPS is provided by $\{NH_T\}$ at a pH < 1 and by $\{SiOH_T\}$ at a pH > 10.

We set

$$P = exp\left(-\frac{zF\psi_0}{RT}\right), \quad \{H^+\} = [H^+] \times P^{-1} \quad \text{(S24)}$$

For a positive surface:

$$K_N^{int} = \frac{\{H^+\}\{NH_3\}}{\{NH_4^+\}} = [H^+] \times P^{-1} \times \frac{\{NH_T\} - \{SiOH_T\} + \{SiO^-\}}{\{SiOH_T\} - \{SiO^-\}} \quad \text{(S25)}$$

For a negative surface:

$$K_{Si}^{int} = \frac{\{H^+\}\{SIO^-\}}{\{SiOH\}} = [H^+] \times P \times \frac{\{NH_T\} - \{NH_4^+\}}{\{SiOH_T\} - \{NH_T\} + \{NH_4^+\}} \quad \text{(S26)}$$

In the main text, in the **Surface electrical properties** section, $\{X^+_N\}$ is noted as the net charge at 10 > pH > pH_{pzc} and $\{X^-_{Si}\}$ as the net charge at 1 < pH < pH_{pzc}.

At pH > pH_{pzc},

$$\{X_N^+\} = \{SiO^-\} - \{R - NH_4^+\} = \{SiOH_T\} - \{R - NH_4^+\} \quad (S27)$$

$$\{R - NH_4^+\} = \{SiOH_T\} - \{X_N^+\} \quad (S28)$$

At pH < pH_{pzc},

$$\{X_{Si}^-\} = \{R - NH_4^+\} - \{SiO^-\} = \{NH_T\} - \{SiO^-\} \quad (S29)$$

$$\{SiO^-\} = \{NH_T\} - \{X_{Si}^-\} \quad (S30)$$

Then,

$$K_N^{int} = \frac{\{H^+\}\{NH_3\}}{\{NH_4^+\}} = [H^+] \times P^{-1} \times \frac{\{NH_T\} - \{SiOH_T\} + \{X_{Si}^-\}}{\{SiOH_T\} - \{X_{Si}^-\}};\ \ pH > pH_{zpc} \quad (S31)$$

$$K_{Si}^{int} = \frac{\{H^+\}\{SiO^-\}}{\{SiOH\}} = [H^+] \times P \times \frac{\{NH_T\} - \{X_N^+\}}{\{SiOH_T\} - \{NH_T\} + \{X_N^+\}};\ \ pH < pH_{zpc} \quad (S32)$$

Thus,

$$\frac{1}{[H^+] \times P^{-1}} = \frac{\{NH_T\}}{K_N^{int}} \times \frac{1}{\{SiOH_T\} - \{X_{Si}^-\}} - \frac{1}{K_N^{int}}\ ; @pH > pH_{zpc} \quad (S33)$$

$$[H^+] \times P = \{SiOH_T\} \times K_{Si}^{int} \times \frac{1}{\{NH_T\} - \{X_N^+\}} - K_{Si}^{int}; @pH < pH_{zpc} \quad (S34)$$

NH_T is the total concentration of active amino sites in mol·L^{-1}.

$SiOH_T$ is the total concentration of active silanol sites in mol·L^{-1}.

The surface charge per liter of solution (mol·L^{-1}) can be calculated using the following equation:

$$\{X\} = \frac{n_x \times w}{V} \quad (S35)$$

where

w is the weight of the sample in g;

V is the volume of the solution in L.

Thus, the surface electrical properties of materials can be calculated.

SCFM calculation:

From the work of Healy (1972) and Park and Huang (1989),

$$\Delta G^0_{coul} = z_i F \times \Delta\psi_x \quad \text{(S36)}$$

At a distance x from the surface of the adsorbent, the potential drop at this point is ψ_x; then,

$$Q = exp\left(\frac{zF\psi_0}{2RT}\right) \quad \text{(S37)}$$

$$x = r_{UO_2} + 2r_w \quad \text{(S38)}$$

$$\Delta\psi_x = \frac{2RT}{zF} \times \ln\left\{\frac{(Q+1)+(Q-1)\times e^{-\kappa x}}{(Q+1)-(Q-1)\times e^{-\kappa x}}\right\} \quad \text{(S39)}$$

r_{UO_2} is the radius of UO_2 (0.64nm), and r_w is the radius of a water molecule (0.138nm). The calculation of the solvation energy ΔG^0_{solv} was employed by James and Healy (1972). They successfully fitted the calculated data and the experimental results for Fe(III), Cr(III), Co(II), and Ca(II) with SiO_2:

$$\Delta G^0_{solv} = \left(\frac{z^2e^2N}{16\pi\varepsilon_0}\right)\left(\frac{1}{r_{UO_2}+2r_w} - \frac{r_{UO_2}}{2\left(r_{UO_2}+2r_w\right)^2}\right)\left(\frac{1}{\varepsilon_{int}} - \frac{1}{\varepsilon_{bulk}}\right) + \left(\frac{z^2e^2N}{32\pi\varepsilon_0}\right)\left(\frac{1}{r_{UO_2}+2r_w}\right)\left(\frac{1}{\varepsilon_{solid}} - \frac{1}{\varepsilon_{int}}\right)$$

(S40)

$$\varepsilon_{int} = \left\{\frac{\varepsilon_{bulk}-6}{1+1.2\times10^{-17}(d\psi/dx)^2_x}\right\} + 6 \quad \text{(S41)}$$

$$\frac{d\psi}{dx} = -2\kappa x \frac{RT}{zF} sinh\left(\frac{zF\times\Delta\psi_x}{2RT}\right) \quad \text{(S42)}$$

where κ^{-1} is the value of the diffusion layer thickness (m); e is the polar molecule ionic charge ($1.602\times10^{-19}C$); ψ is the potential at distance d (V); d is the distance from the particle surface (nm); ε_0 is the permittivity of empty space (8.85×10^{-12} F/m); ε_r is the relative permittivity of water (*78 @ 25 ℃*); z_i is the electrolyte charge z_+ versus z_-; in this case, $z_i = 1$; T is the temperature (K); N is

Avogadro's number (6.023×10^{23}); R is the molar gas constant ($R = 8.314\ J\cdot mol^{-1}\cdot K^{-1}$); and F is the Faraday constant ($F = 96485 C\cdot mol^{-1}$).

Eq (S41) was proposed by Laidler and Sacher (1954). James and Healy (1972) applied the dielectric constant at the interface as a constant value of 6 instead of n_∞^2 from the original equation.

The lateral interaction ΔG^0_{lat} is the interaction between the adsorbed ions. In this case, $X^+:U^{ads}_{Max}$ is low (Table 2), demonstrating light surface loading (Huang et al. 1987). Thus, $\Delta G^0_{lat} \approx 0$ for light surface loading.

For an individual species,

$$\Delta G^0_{ads} = 2.303 \times R \times T \times pK_i^s \tag{S43}$$

Based on the work of Stumm (1992), Hao and Huang (1986), James and Parks (1982), and Weng et al. (2001), during the adsorption process, the chemical energy ΔG^0_{chem} between a pair of interacting ions is constant.

When we reform eq(5) in the main text, it yields:

$$\Delta G^0_{chem} = \Delta G^0_{ads} - \Delta G^0_{coul} - \Delta G^0_{solv} - \Delta G^0_{lat} \tag{S44}$$

Table S2. U(VI) hydrolysis reactions

Equation	-Log K	Ref.
$UO_2^{2+} + OH^- \rightarrow UO_2OH^+$	5.25±0.24	[21]
$2UO_2^{2+} + 2OH^- \rightarrow (UO_2)_2(OH)_2^{2+}$	5.62±0.06	[21]
$3UO_2^{2+} + 4OH^- \rightarrow (UO_2)_3(OH)_4^{2+}$	11.90±0.30	[21]
$3UO_2^{2+} + 5OH^- \rightarrow (UO_2)_3(OH)_5^{+}$	15.55±0.12	[16]
$4UO_2^{2+} + 7OH^- \rightarrow (UO_2)_4(OH)_7^{+}$	21.90±1.00	[16]
$UO_2^{2+} + 2OH^- \rightarrow UO_2OH_2(aq)$	12.15±0.17	[21]

$3UO_2^{2+} + 7OH^- \rightarrow (UO_2)_3(OH)_7^-$	32.20±0.80	[21]
$UO_2^{2+} + 3OH^- \rightarrow UO_2(OH)_3^-$	20.70±0.42	[21]
$UO_2^{2+} + 4OH^- \rightarrow UO_2(OH)_4^{2-}$	31.90±0.33	[21]
